# GeneSNAKE: a Python package for simulation of gene regulatory networks and perturbation-induced expression data

**DOI:** 10.1093/bioadv/vbag039

**Published:** 2026-02-06

**Authors:** Thomas Hillerton, Anton Björk, Nils Lundqvist, Erik K Zhivkoplias, Mateusz Garbulowski, Erik L L Sonnhammer

**Affiliations:** Department of Biochemistry and Biophysics, Stockholm University, Science for Life Laboratory, Solna 17121, Sweden; Department of Biochemistry and Biophysics, Stockholm University, Science for Life Laboratory, Solna 17121, Sweden; Department of Biochemistry and Biophysics, Stockholm University, Science for Life Laboratory, Solna 17121, Sweden; Department of Biochemistry and Biophysics, Stockholm University, Science for Life Laboratory, Solna 17121, Sweden; Department of Biochemistry and Biophysics, Stockholm University, Science for Life Laboratory, Solna 17121, Sweden; Department of Biochemistry and Biophysics, Stockholm University, Science for Life Laboratory, Solna 17121, Sweden

## Abstract

**Motivation:**

Understanding how genes interact with and regulate each other is a key challenge in systems biology. One of the primary methods to study this is through gene regulatory networks (GRNs). The field of GRN inference faces many challenges, which necessitate effective tools for evaluating inference methods. Data that corresponds to a known GRN, from various conditions and experimental setups is necessary for this purpose, which is only possible to attain via simulation. However, most existing tools for GRN-based simulation are limited either in network or data properties, with few or no options to modify these properties.

**Results:**

We present GeneSNAKE, a Python package designed to allow users to generate biologically realistic GRNs and expression data for benchmarking purposes. GeneSNAKE improves on previous work by providing a unique combination of modules, allowing users to control a wide range of GRN and data properties. It provides full control of the noise level, several noise models, full control of the perturbation design, and a wide range of pre-defined perturbation schemes. For benchmarking, GeneSNAKE offers several functions both for comparing network similarity, and properties in data and GRNs. These functions can further be used to study properties of biological data to produce simulated data with more realistic properties.

**Availability and implementation:**

GeneSNAKE is an open-source, comprehensive simulation and benchmarking package with powerful capabilities that are not combined in any other single package. Thanks to the Python implementation, it can be extended and modified by users. The tool is available at: https://bitbucket.org/sonnhammergrni/genesnake/

## 1 Introduction

Understanding how genes interact with and regulate each other has long been one of the key challenges in systems biology. One of the primary methods to study this is through gene regulatory networks (GRNs). The field of GRN inference however still faces many challenges, such as the complexity of gene regulation, high noise levels of expression data, and that a majority of the true regulatory interactions are unknown making any finding hard to verify. With these challenges in mind, a large number of tools have been developed to tackle the issue of GRN inference (GRNI), using a variety of statistical and machine learning approaches with a focus on different types of data and experimental approaches to capture the regulatory connections (Sanguinetti and Huynh-Thu 2019). Therefore, a large number of methods, all with slightly different focus, necessitates effective tools for evaluating inference methods. For this purpose, data that corresponds to a known GRN and is derived from cells at similar conditions and cell cycle phase yet from various experimental conditions and setups would be required. A key issue with this approach arises here in the form of limited coverage of experimentally validated GRNs—for instance, the human GRN in TRRUST is limited to 8444 regulatory interactions for 800 transcription factors (Han *et al.* 2018). Due to this, obtaining data for a known GRN to evaluate GRNI methods is today impossible due to the excessive number of experiments that would be required both to properly determine the exact underlying GRN and obtaining informative data for meaningful comparison. This is often reflected in benchmarking papers where methods tested on real data often have a correctness so low that it can often not be distinguished from random performance (Marbach *et al.* 2012). Because of this the GRN field traditionally relies on synthetic data for developing and evaluating methods (Greenfield *et al.* 2010, Madar *et al.* 2010).

The need for synthetic data has led to a number of popular tools for gene expression simulation. The most popular option for simulators is the GeneNetWeaver (GNW) tool published by Schaffter *et al.* (2011). However, both earlier and later simulators (Van den Bulcke *et al.* 2006, Li *et al.* 2009, Tjärnberg *et al.* 2017, Pratapa *et al.* 2020, Dibaeinia and Sinha 2020, Herbach 2023, Zinati *et al.* 2024, Li *et al.* 2025, Harlapur *et al.* 2025) have been developed in an attempt to address the lack of data for GRNs. While these tools often perform well for the task at hand, they often suffer from being overly focused on a single issue of the data generation. For example, GNW focuses heavily on selecting a biologically realistic GRN to generate the data from. This, however, comes at the expense of having very limited control over data properties and experimental design. The tool GeneSPIDER (Tjärnberg *et al.* 2017, Garbulowski *et al.* 2024) allows the user to control the noise level and condition number when generating data, but can only simulate data at steady state. Another example is the BoolODE model developed by Pratapa *et al.* (2020) for simulating single-cell gene expression data given a GRN, but it does not have built-in functionality for the user to generate a GRN, and is limited to using Boolean GRNs. While this specialization is not necessarily negative it does limit the applicability of the method as there is still no consensus in the GRN field of what properties are most important for GRNI. In the case of GNW, knockdown perturbations are limited to a fixed strength of 0.5, and for BoolODE no perturbation design can be specified. These shortcomings create a highly limited view when used for testing GRNI methods, as not all biological experiments follow a similar design or degree of perturbation. Another tool SERGIO (Dibaeinia and Sinha 2020) allows versatile perturbation designs and noise modeling, yet can only simulate data from networks with a directed acyclic graph topology. This network topology, while computationally easy to work with, has been shown to not be biologically realistic, as multiple motifs and feedback cycles are present and play key roles in real GRNs (Shen-Orr *et al.* 2002, Milo *et al.* 2002, Zhivkoplias *et al.* 2022, Massri *et al.* 2023). Most of these methods additionally lack the ability to generate GRNs, meaning they will only work if the user has a suitable GRN model prepared separately, especially recent methods focusing on single-cell data such as BoolODE (Pratapa *et al.* 2020), SERGIO (Dibaeinia and Sinha 2020), and scMultiSim (Li *et al.* 2025). For several simulators, there are also limitations with the applied noise, often being confined to a single noise model with limited or no control of the noise level. Flexible noise modeling is important given that assumptions about noise are seldom applicable to real data sets. Finally, a recurring issue with many simulators is that the software is hard to access either due to a lack of documentation, the code not being available, or the simulator being written in a language rarely used in bioinformatics. This is something that can either discourage or completely prevent the usage of a simulator for groups working with developing inference tools or studying GRNs.

**Figure 1:**
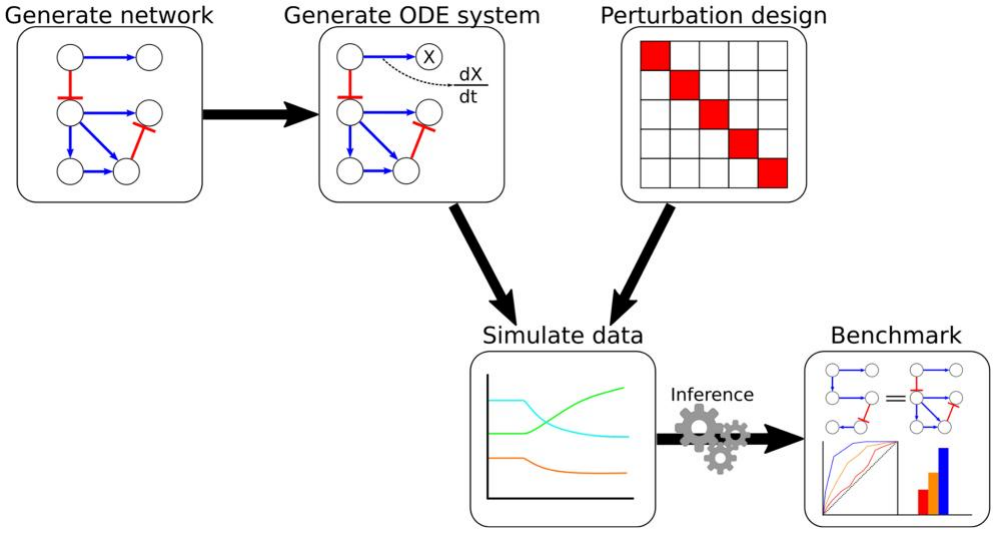
GeneSNAKE workflow. GeneSNAKE can generate GRNs, and from these, an ODE system that is used to generate time series and steady-state gene expression data following a large number of possible perturbation designs. The generated GRNs and simulated data feed into a benchmarking module to compare GRNs inferred from the data with the GRN used to generate it.

Here we present Generation and Simulation of Networks and datA pacKagE (GeneSNAKE), a python package for generation of synthetic data that follows the dynamics specified in a GRN model. GeneSNAKE builds on previous works relying on a commonly used ordinary differential equation (ODE) model but offers further functionality aimed at improving GRN generation, experimental design, and dynamic user-defined data properties, see Fig. 1. To address weaknesses of previous methods a focus has been placed on ensuring a balance between biological realism and flexibility, see Table 1. This has been ensured both by creating functions that generate GRNs and expression data that are biologically feasible and by allowing users to modify most parameters to control the generation in great detail. Further to allow for robust and varied experimental design when it comes to perturbing the system, GeneSNAKE offers a variety of predefined perturbation designs that can be used to generate data. GNW only allows perturbations at fixed strengths for knockdown (50%) and knockout (100%), respectively. For maximum flexibility, GeneSNAKE allows the user to choose perturbation strength per gene anywhere between 100% knockdown and infinite overexpression to simulate data with any experimental design. Finally, to offer as much flexibility as possible, GeneSNAKE includes multiple noise models that capture biologically relevant noise in the data and allow the users to customize the noise degree such that various experimental conditions can be approximated.

**Table 1:** Comparison of selected tools for simulating GRN-based gene expression data.

Tool	GeneSNAKE	GeneSPIDER	GeneNetWeaver	BoolODE	SERGIO	scMultiSim
Authors	Hillerton *et al.* (2022)	Tjärnberg *et al.* (2017)	Schaffter *et al.* (2011)	Pratapa *et al.* (2020)	Dibaeinia and Sinha (2020)	Li *et al.* (2025)
Programming language	Python	Matlab	Java	Python	Python	R
Distribution	Source code	Source code	Executable program	Source code	Source code	Bioconductor
GRN generation	Internal with flexible topology and motif properties	Internal with limited topology properties	Loads external GRN	No	No	No
Data simulation	Dynamic ODE	Linear ODE approximation	Dynamic ODE	Dynamic ODE	Dynamic SDE	Dynamic kinetic model
Noise models	Gaussian multiplicative Log-normal multiplicative Single-cell sequencing like	Gaussian additive	Gaussian and log-normal additive. SDE model multiplicative Microarray-like	SDE model multiplicative Dropout model	Single-cell-like noise model with cell count and drop out accounted for Technical noise from user provided data	Intrinsic noise in simulation model Single cell seq like technical noise
Perturbation strength and design	Tunable and customizable and pre-defined designs	Tunable and customizable design	Fixed with a single gene or all-genes design. Only knockout/down	Stochastic with all-genes design	Tunable and customizable design	Tunable and customizable design via the GRN kinetics
Last updated	2025	2024	2014	2022	2020	2025

## 2 Methods

### 2.1 Generating GRNs

GeneSNAKE simulates interactions between genes based on a GRN ensuring that any change in the system follows the dynamics described in the GRN. Thus it is important to have access to GRNs with different properties and sizes to simulate data from. Users can input any network to the algorithm in the form of an edge list or adjacency matrix. GeneSNAKE supports the generation of biologically realistic GRNs using the FFLatt algorithm that ensures a motif composition typical for biological GRNs (Zhivkoplias *et al.* 2022). It does this by employing a motif-based preferential attachment algorithm with a power-law kernel, aiming to reproduce the feed-forward loop (FFL) motif occurrence observed in literature-based biological GRNs (Milo *et al.* 2002). It also preserves important topological properties such as scale-free topology, sparsity, and average in- and out-degree per node. The FFL-enriched network is generated with attachment rules and predetermined probabilities that control for the emergence of new FFL motifs at every iteration. The algorithm also allows for depletion of other three-node motifs, such as cascades, downlinks, uplinks, and cycles. GeneSNAKE further supports integration of any Networkx graph. Networkx is a commonly used graph handler in Python (Hagberg *et al.* 2008). To obtain an overview of installation and how-to guidelines we encourage the user to visit the GeneSNAKE tutorial website: https://sonnhammer-tutorials.bitbucket.io/genesnake.html. The tutorial includes step-by-step examples, as well as descriptions of the GeneSNAKE parameters.

### 2.2 Modeling gene expression as a molecular system

Model assumptions must be made on how the real system behaves and how closely the real system must be mimicked to make it useful. For GeneSNAKE several assumptions are made based on how genes are expressed and regulated. The most fundamental assumption made when creating the GeneSNAKE model is that gene expression works like a probabilistic molecular system that exists freely in solution. In practical terms, this means that gene expression is described as a sigmoid function, where activation and repression of a target gene is dependent on the concentration of the regulator gene, through a Hill equation where both minimum and maximum expression is bounded. The model assumes that transcription and translation occur in the same space, hence no molecular transport is required. This assumption is based on findings indicating that the time for translocation of mRNA and proteins is significantly lower than the half-life of molecules in the cytoplasm, <10 minutes and several hours, respectively, in mice (Audibert *et al.* 2002). Further, the model assumes that the ribosomal concentration is always higher than the mRNA concentration and thus the relation between protein concentration and mRNA concentration for a given gene can be described by a linear differential equation where each mRNA molecule for a given gene is transcribed once per time step. This relationship ensures that the protein expression follows a similar sigmoid distribution as the mRNA, ensuring that the expression is bounded within the system. Finally, degradation of both protein and mRNA is modeled as a linear function based on a constant degradation rate for each molecule, with the assumption that the degradation systems for protein and mRNA always have more capacity than their maximum expression in the system. As degradation rates vary between different genes, we assign a random value to the degradation constant (*λ*) to make some genes degrade faster than others. The degradation rate for proteins is assigned to a lower value than for mRNA molecules on average to simulate the longer average half-life of proteins (Faith *et al.* 2007). With these assumptions, gene expression can be modeled with the two-step ODE model developed for the DREAM3 challenge (Marbach *et al.* 2010). This model was selected as the basis for GeneSNAKE due to its frequent and well-established use in the GRN field. It is also employed by GeneNetWeaver (Schaffter *et al.* 2011) and BoolODE (Pratapa *et al.* 2020), two popular simulation tools in the GRNI field.

### 2.3 Ordinary differential equations describing gene regulatory interactions

Using the above described assumptions on how genes are expressed and interact within a GRN it becomes possible to simulate the dynamic behavior in the system using two ODEs [Equation (1) for mRNA and Equation (2) for protein]. The change in mRNA concentration [Equation (1)] for a given gene (*x_i_*) is described as the addition of new molecules, calculated as the max transcription rate (*m_i_*) times the protein concentration of each regulatory gene (*y*_reg_) given by the function *f*(*y*_reg_), minus the degradation of existing molecules, calculated as the degradation constant *λ*^mRNA^ times the current mRNA concentration *x_i_*.


(1) $$\frac{dx_{i}}{dt}=m_{i}\cdot f(y_{\mathrm{reg}})-\lambda_{i}^{\mathrm{mRNA}}\cdot x_{i}$$

(2) $$\frac{dy_{i}}{dt}=r_{i}\cdot x_{i}-\lambda_{i}^{\mathrm{prot}}\cdot y_{i}$$

For the GeneSNAKE model, *m_i_* is set to 1 for all unperturbed genes in the system and *λ_i_*^mRNA^ is randomly assigned a value from a uniform distribution between 0.2 and 0.7. The activation function *f*(*y*_reg_) depends on the protein concentration (*y*) of each regulatory gene and consists of a Hill function [Equation (3)]. As the GeneSNAKE model assumes that all interactions occur through protein-mediated interactions, the change in protein concentration (*y*) over time is also modeled [Equation (2)]. This extra step allows the model to capture the time lag between a change in mRNA concentration and the corresponding change in protein concentration. The change in protein concentration [Equation (2)] is described by the number of new proteins translated, calculated as the max translation rate (*r_i_*) times the mRNA concentration (*x_i_*), minus the number of degraded protein molecules, calculated as the degradation rate *λ_i_*^prot^ times the number of existing protein molecules. *λ_i_*^prot^ is randomly assigned to a value between 0.2 and 0.4.

To allow for complex interactions within the system, GeneSNAKE models regulations where two or more regulators interact, using an additive Hill function, shown for two regulators in Equation (4). For the combinatory activation, the total activation is given by the effect from each regulator plus that of the combined, multiplicative effect of the regulators acting together. To model the combinatory effect, a combination factor *P* is used. In GeneSNAKE *P* is assigned to either 1 or 0, indicating either a combination that requires all participants, simulating an AND logic, or no combinatory effect, simulating an OR logic. In Equation (5) this is done by a multiplication operator to get the AND logic, and addition to get OR. The combinatory effect is in GeneSNAKE limited such that all participants must be either activating or inhibiting, and no combinations can be made of regulators with opposing effects. For all of the activation/inhibition functions [Equations (3)–(5)] GeneSNAKE uses the same parameters; α describes the regulatory effect in how strongly the target expression is affected, *k* is the dissociation constant, roughly equivalent to how likely it is that a regulator molecule does not have an effect, and *n* is the Hill coefficient, describing how quickly the regulator changes the expression. In GeneSNAKE these parameters are randomly assigned: *k* is set to a value between 0.2 and 0.6, and *n* to an int value between 2 and 4 (traditional range of values that will give a hill function). α is a special case being assigned values based on network properties. If the edge suggests a strong interaction (defined as an edge value greater than the median absolute edge weight in the network) then α is assigned a value between 0.6 and 1.0. If instead the edge is a weak interaction (edge value less than or equal to the median absolute edge weight) then α is assigned to a value between 0.4 and 0.8. Finally, if there is a collaborative interaction between two or more regulators (*P* = 1) each regulator is assigned a weak α, i.e. 0.2–0.4, on their own and a strong α_3_, i.e. 0.7–1.0, for the combination.


(3) $$f(y_{\mathrm{reg}})=\alpha \frac{{\left(\frac{y_{\mathrm{reg}}}{k}\right)}^{n}}{1+{\left(\frac{y_{\mathrm{reg}}}{k}\right)}^{n}}$$

(4) $$f(y_{1},y_{2})=\frac{\alpha_{1}V_{1}+\alpha_{2}V_{2}+\alpha_{3}PV_{1}V_{2}}{1+V_{1}+V_{2}+PV_{1}V_{2}}$$

(5) $$V_{i}={\left(\frac{y_{i}}{k_{i}}\right)}^{n_{i}}$$

By enforcing a strict mRNA to protein to regulatory effect, the model can simulate dynamic time-delayed chains of reactions following the edges of the GRN, allowing for simulation of gene expression in both time series and steady-state experiments. The dynamic non-linear nature of the model allows for simulation of both complex and simple relationships between genes with multiple regulators collaborating or acting independently as desired by the user.

For ease of implementation and future-proofing, GeneSNAKE relies on the Python package Scipy (Virtanen *et al.* 2020) for solving ODE systems. Specifically, it uses the solve_ivp function from Scipy relying on LSODA, a Fortran-based ODE solver (Hindmarsh 1983). To ensure that the solution is robust the error tolerance was set to 1 × 10^−8^ to minimize errors in the simulation.

### 2.4 Simulating perturbations

A key requirement for inferring GRNs from gene expression is the perturbation of the system—to capture the system there must be a change that can be detected and explained. To simulate perturbations, GeneSNAKE modifies the max transcription rate parameter of Equation (1) (*m_i_*) in the following way:


(6) $$m_{i}=m_{i}^{\mathrm{input}}\cdot (1+\mathrm{pert})$$

where pert is a value between −1 and infinity, and *m_i_*^input^ is the original max transcription rate value used to obtain the control expression, by default 1. This models the perturbation by setting pert, the wanted change, to a non-zero value. For instance, pert = −0.8 gives an 80% reduction of the original transcription rate in the control. The ability for the user to vary the strength of the perturbation allows GeneSNAKE to more closely capture the effect of real experiments where perturbation strength can vary highly, either by design (Jost *et al.* 2020) or due to the experimental method, e.g. siRNA, CRISPRi, or overexpression. Several perturbation schemes have been proposed in the literature, but it is not clear which one is the best from a GRNI perspective. Because of this, GeneSNAKE features a wide variety of options for the generation of perturbation schemes as well as support for using custom perturbation designs. Currently, GeneSNAKE supports the perturbation schemes:

- Single gene—Each gene in the system is perturbed one by one so that all genes in the system are perturbed once.
- Combinatory—Multiple genes are perturbed in each experiment in such a way that each gene is perturbed roughly an equal amount of times across all experiments. The number of perturbed genes per experiment is determined by the user.
- Targeted combinations—Multiple genes are perturbed in each experiment and combinations are selected from the GRN so that genes regulating the same target are perturbed in the combinations. The number of perturbed genes per experiment is determined by the user.
- All genes—All genes in the system are perturbed with a random but small effect, either positive or negative, to simulate global perturbation experiments such as cell medium or temperature changes.
- Specific genes—A single or multiple genes are perturbed in each experiment based on an input list of gene names and gene combinations to perturb. Only those genes in the list will be perturbed.

For all of the perturbation schemes, excluding single perturbation, the number of perturbed genes per experiment is only limited by the total number of genes and the number of simultaneously perturbed genes. For targeted combinations, the algorithm will generate combinations with fewer genes in them if it is not possible to find combinations with the requested number of genes. This is to ensure that as many unique experiments as possible are created. It makes sure that each experiment has a unique perturbation scheme, except for when using the random perturbation scheme where experiments can be identical. The experiment parameters are of particular use for the combinatory and targeted perturbation scheme where possible combinations of genes will be selected at random until the maximum number of experiments have been achieved. Utilizing these schemes allows for high flexibility in the experimental design while automating its creation allowing for quick simulations of complex studies in a user-friendly manner. To make more elaborate experimental designs it is possible to merge multiple perturbation schemes and use this to simulate data, for example, to use a design of single gene perturbation of all genes along with a set of interesting combinations to be simulated.

### 2.5 Noise modeling

Noise is one of the hardest obstacles for GRNI. Unfortunately, the exact nature of biological noise is today undetermined, and therefore GeneSNAKE offers several noise models as well as an option for the user to input either a noise function or a matrix of noise to add. Currently, three noise models are available in GeneSNAKE. The first one is a Gaussian noise model where the signal in each experiment is multiplied with noise drawn from a Gaussian distribution. Second, a log-normal noise model, previously shown to model microarray noise well (Tu *et al.* 2002, Stolovitzky *et al.* 2005, Schaffter *et al.* 2011), where log-normal distributed noise is multiplied with the signal. Finally, a single-cell sequencing noise model based on a zero-inflated negative binomial noise distribution. This model uses a simulated coverage, gene length, error rate, and dropout rate to model biases in single-cell sequencing experiments (for details see Supplementary Text 2 and Fig. 1, available as supplementary data at *Bioinformatics Advances* online).

### 2.6 Simulating an experiment

Using the above described ODE, perturbation scheme, and noise models GeneSNAKE simulates any number of experiments for measuring gene expression. Despite all the different options, the simulation of experiments is consistent, i.e. each simulation starts with the creation of a control expression of the unperturbed system at a steady-state. The initial steady-state is determined by simulating the data from a random start until a steady-state is found. Steady-state is here determined by applying a multidimensional root-finding test ensuring that the change in the system is small enough, below 0.00001 for all genes, that we can consider it to have converged to a steady-state. As a steady-state is not always possible the run will be ended if a steady state is not found after 10 000 steps. GeneSNAKE then simulates each experiment in the perturbation design matrix by applying the selected perturbation to the gene expression and solving the ODE system using the control values as the starting point. The ODE system is then solved either for a given time span or until a new steady-state is detected by the Kalman filter in the system, depending on if the user requested generating time series data with multiple time points or steady-state data with only a single time point as output.

Once the simulation is complete, GeneSNAKE returns the experimental and control data along with the used noise for each generated time point. For time series data, to create biologically realistic simulations only a limited number of timepoints is returned, for which the exact number can be set by the user. The time points are selected either in a uniform linear distribution across the whole simulation time or in a log distribution with a diminishing number of timepoints as the time increases. For steady-state experiments, only a single time point is returned at the stopping point determined by a point where no gene or protein has moved more than a tolerance value for at least two time steps. It is also possible to use this as a stopping point for time series data to ensure that all the time points are located in the shifting part of the experiment rather than in steady-state areas. If no steady-state can be found due to a cyclic behavior in the system, steady-state data will instead be defined as the last time step in the model.

### 2.7 GRN inference

Figure 3 shows how inference success varies with SNR level in GeneSNAKE simulations. For this investigation, we used LSCO (Tjärnberg *et al.* 2015), LSCON (Hillerton *et al.* 2022), LASSO (Tibshirani 1996, Bonneau *et al.* 2006, Tjärnberg *et al.* 2013), two variants of Z-score (Prill *et al.* 2010), and GENIE3 (Huynh-Thu *et al.* 2010). For method descriptions, see Supplementary Text 3, available as supplementary data at *Bioinformatics Advances* online. These methods were selected for being well-known and conveniently accessible, to demonstrate that the data produced by GeneSNAKE is useful for investigating the effect of noise on GRN inference accuracy.

### 2.8 Benchmarking

For GRN inference performance evaluation, GeneSNAKE computes and plots receiver operating characteristic (ROC) curves, precision recall (PR) curves, and the areas under (AU) them, resulting in AUROC and AUPR values. Additional available metrics are listed in Supplementary Text 1, available as supplementary data at *Bioinformatics Advances* online. For comparability across methods and situations, the curves are extended when predicted networks are incomplete. For ROC curves, the standard linear extension to (1, 1) is applied. For PR curves, the extension is done according to the DREAM 2 challenge performance evaluation (Stolovitzky *et al.* 2009). For both the ROC and PR cases, the extensions are equivalent to randomly guessing edge existence for the missing part of the predicted network, which is trivial to perform, and thus guaranteed to be possible to realize. GENIE3 never infers self-regulatory interactions for genes. To avoid excessively penalizing this, the benchmarking of GENIE3 predictions in this publication excludes self-regulatory interactions. For all other methods, they are included. All parameters used for all aspects of the method performance evaluation are included with the publication reproduction code.

### 2.9 Processing of ENCODE reference data

shRNA-seq perturbation experiments from the ENCODE project were used as reference for properties of simulated data. Perturbation data from the K562 and HepG2 cell lines and corresponding unperturbed control samples were downloaded from ENCODE (ENCODE Project Consortium 2012). Gene expression in terms of transcript abundance was quantified using pseudo alignment with Salmon (Patro *et al.* 2017). To calculate log2 fold-change (log2FC) values, perturbation-induced expression values were compared to the controls, and technical replicates were handled by averaging. Where multiple perturbations targeted the same gene, they were averaged to a single log2FC value. Code and detailed description of the data processing is available in the publication reproduction code repository (Data Availability section).

## 3 Results

**Figure 2:**
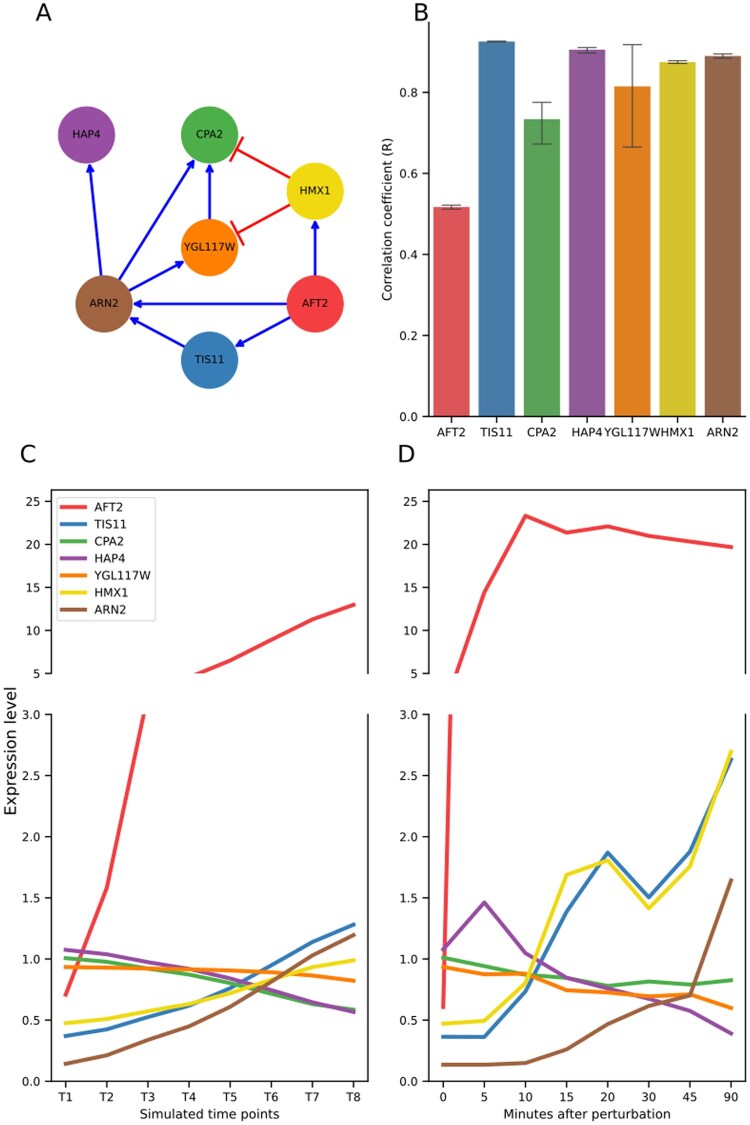
Correlation between GeneSNAKE simulation and experimental data. (A) Subset GRN of the transcription factor AFT2 and downstream genes in the system experimentally characterized by Hackett *et al*. (2020). (B) The Pearson correlation for each gene between simulated and experimental data. The error bars indicate 95% confidence interval over 5 runs. (C) Expression levels simulated by GeneSNAKE over time using Hackett’s GRN model. The simulation was run for all genes in the system, but for clarity only the AFT2 subset GRN genes are shown. The first 8 time points of 10 were selected to match the experiment by Hackett *et al*., which was not performed until steady state. (D) Expression levels measured experimentally in yeast for the subset GRN genes over time after artificial induction of AFT2. Note that the upregulation of AFT2 in the experimental data is faster than in the simulated data due to the use of an artificial induction system for rapid overexpression.

GeneSNAKE is a simulation tool that relies on an ODE model to capture the dynamic behavior of gene regulatory interactions. Building on previous work, GeneSNAKE’s ODE system shares many features with other simulators. Although this methodology has often been used to simulate data for GRN inference, only limited testing has been done to assess how well it models the dynamics of a real system. To address this and demonstrate that GeneSNAKE is capable of capturing the complex dynamics observed in biological experiments, we tested how well the simulated data correlates with experimental data derived from work by Hackett *et al.* (2020). This experiment studied the effect of overexpressing selected transcription factors in Saccharomyces *cerevisiae*. We tested whether GeneSNAKE can capture the same effect by simulating the same experiments and measuring how well the simulated data correlates with the experimental data. Figure 2 shows the dynamics and gene-wise correlations for a subset GRN from the Hackett dataset. The subset GRN was selected starting from AFT2 as it has no incoming edges but three outgoing edges, meaning it is not regulated within the total GRN, but regulates several other genes. All genes within two steps downstream of AFT2 in the total GRN were selected for the subset GRN. This small GRN was selected purely for visualization purposes, and the full GRN was simulated to ensure that all dynamics are preserved. It can be seen that while the absolute expression levels are generally not the same, the behavioral trends are similar. The correlation between simulated and measured values are generally high, 0.7–0.9 for all genes except the highly overexpressed AFT2, indicating that the simulation largely follows the trends seen in experimental data. GeneSNAKE’s ODE model dynamics do not allow the instant 20-fold upregulation of AFT2 that the artificial induction experiment yields, hence its correlation was only 0.5.

**Figure 3:**
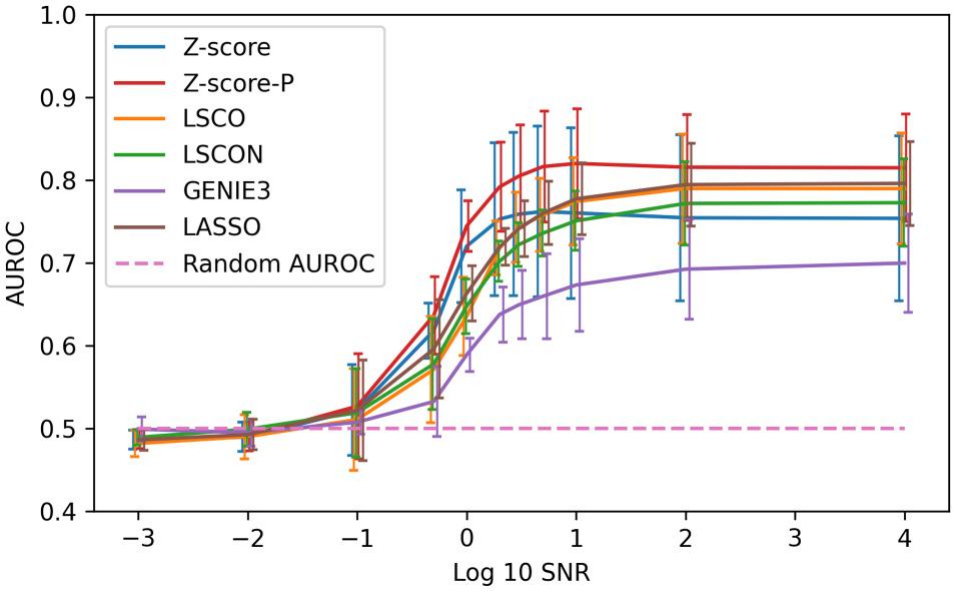
GRN inference accuracy against SNR level, using data produced by GeneSNAKE. Steady-state data with different signal to noise ratio (SNR) levels was simulated from FFLatt GRNs containing 100 genes. Accuracy was measured in terms of area under the receiver operating characteristic (AUROC). Error bars are ±1 standard deviation based on seven different networks and data simulations. The error bars are slightly offset along the *x*-axis for visual clarity. The same SNR levels were recorded for all model types.

**Figure 4:**
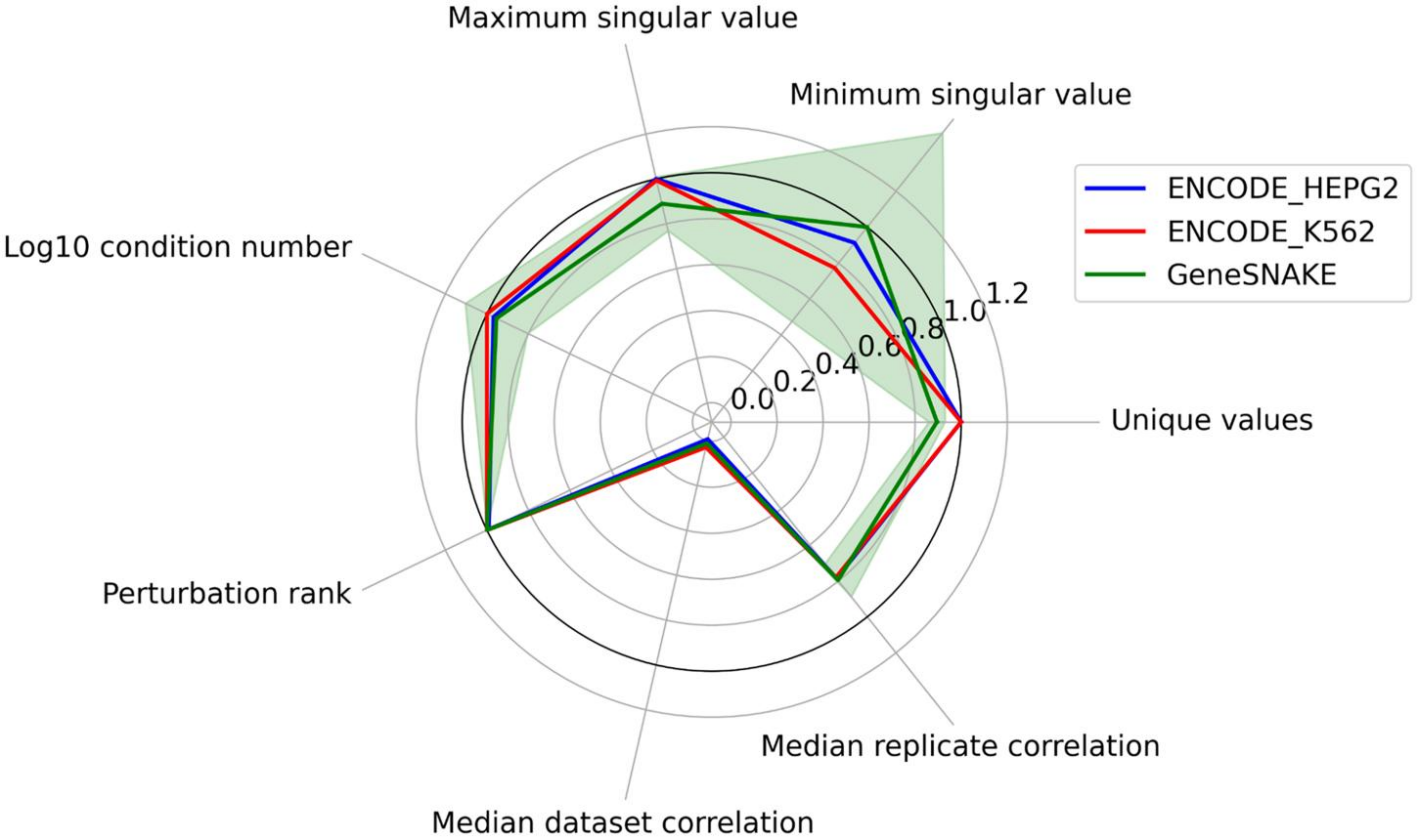
GeneSNAKE can generate realistic perturbation-induced gene expression data. Comparison of data properties from real (ENCODE cell lines K562 and HepG2) and GeneSNAKE simulated data of the same size. All datasets contain log fold changes at steady state after single gene knockdowns. Fraction unique values, median replicate correlation, median data set correlation, and perturbation rank are shown as original values, while log 10 condition number and singular values are shown as the proportion of the respective maximum value among the three datasets. Perturbation rank indicates the specificity of the knock-down to the target gene. A rank of 1 means that the target was most knocked down of all genes. Shown is the median fractional knock-down rank across all genes. The variation of GeneSNAKE data properties is shown as a shaded green area stretching to the mean ± 1 standard deviation based on five network and data simulations.

Next, we wanted to demonstrate that GeneSNAKE can simulate data with a meaningful connection between the underlying GRN and the simulated data. To test this, GRNs were created using the GeneSNAKE GRN generation tool with the FFLatt algorithm. GeneSNAKE was then used to generate an ODE model and simulate single perturbation steady-state data from this network with one perturbation for each gene across three replicates. A range of signal to noise ratio (SNR) levels were applied, going from very high to very low amounts of added noise, where SNR is defined as:


(7) $$\mathrm{SNR}=\frac{s}{\sigma }$$

where *s* is the expression of a given gene in a given experiment which is multiplied with noise sampled from a Gaussian with a mean of 1 and a standard deviation of σ. Figure 3 shows that it is possible to infer accurate networks from GeneSNAKE data, with a clear trend of increasing correctness of the GRN inference methods as the SNR increased. Other metrics show similar trends, Figs 2–4, available as supplementary data at *Bioinformatics Advances* online. The reason that AUROC does not reach 1 even with no added noise can be attributed to the fact that GeneSNAKE’s data generation uses a non-linear model with stochastic components, while the inference methods use linear models. Furthermore, we generated the GRNs with FFLatt that induces a network topology with many feed-forward motifs, which makes the inference task harder. In contrast to the other methods, GENIE3 does not use perturbation information, which explains its lower performance (Seçilmiş *et al.* 2022).

To test how well a GeneSNAKE simulation corresponds to real data, perturbation-based data for cell lines K562 and HepG2 from ENCODE was used (ENCODE Project Consortium 2012). Fold-change expression matrices were computed, where each gene is knocked down in two replicate experiments, for a total of 232 genes and 464 experiments. Networks with the same number of genes were simulated using the FFLatt algorithm, and expression data was generated using the same perturbation design. A comparison of simulated and real data was made using the exploratory data analysis (EDA) tool provided in GeneSNAKE, giving an overview of general data set properties and sample statistics as well as visualizations of the above. Figure 4 shows that GeneSNAKE is able to simulate data which has properties similar to real gene expression data. The simulated data generally matches the real datasets well, although there is a fair bit of variance in the minimum singular value between simulations. Of note, the median replicate correlation is much higher than the median dataset correlation for all three datasets, which suggests that perturbations are successfully applied, and that the data is informative. The low median correlation between observations shows that like the real datasets, GeneSNAKE does not introduce unexpected dependence between observations with different perturbations.

**Figure 5:**
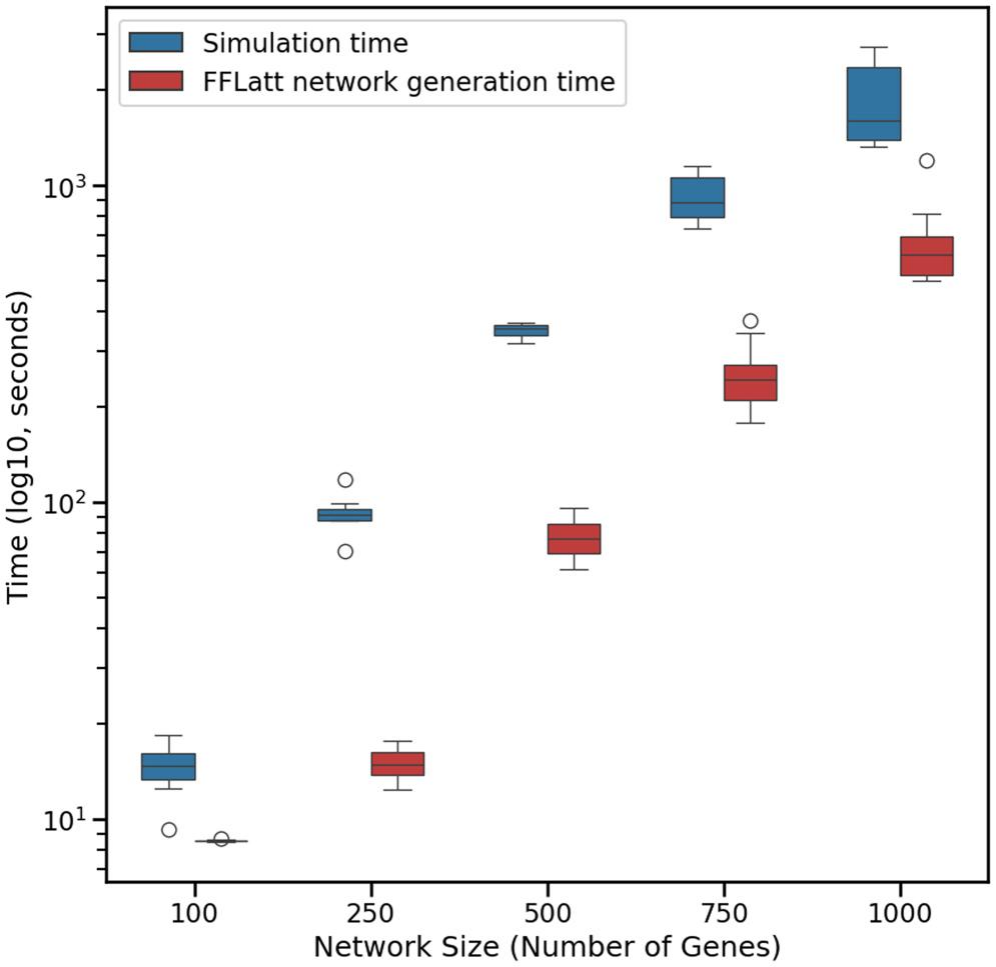
Run time in seconds for GeneSNAKE simulations over varying GRN sizes. Both network generation and simulation were performed on a single core using an AMD Ryzen 9 5950X 16-core processor. Each network generation and subsequent simulation was performed 5 times per size. The analysis shows that as the system grows, solving the ODE system accounts for most of the runtime. The reason for this is that the ODE model by its nature must be solved sequentially, giving it a high time complexity. The simulation was run on a single core on an AMD Ryzen 9 5950X 16-core processor using a network sparsity of about 2.3 links per node and three replicates per datasets with a single perturbation experiment for each gene in the network, creating a genes × genes size dataset. The shaded area marks 1 standard deviation.

Another key feature of a simulation tool is scalability, which we tested by evaluating the run time for increasing GRN size. Figure 5 shows that the runtime for GeneSNAKE’s data generator scales roughly quadratically with the size of the system.

## 4 Discussion

We here present GeneSNAKE, a new Python-based package for generating perturbation-induced gene expression data for a given GRN. The tool is able to generate both GRNs and expression data, as well as perform benchmarking of predicted GRNs compared to ground truth GRNs. We show that the GeneSNAKE ODE model is capable of capturing the same trends over time that are observed in experimental data using the same underlying network, as well as generating data with properties similar to biological data. We also show that the generated data is related to the original GRN and that the noise models can add meaningful noise, allowing developers to explore how different data qualities affect their inference method. GeneSNAKE’s main novelties are the customizable perturbation model, a wide range of perturbation schemes, control of the noise level, and several noise models, in an open-source Python package.

GeneSNAKE functionality covers all the components needed for *in silico* study of GRN inference, see Fig. 1. It is modular by design and uses simple and intuitive data structures at the interfaces between components. Thus, it is easy to substitute any step with other Python software to combine GeneSNAKE with other tools. For example, the GENIE3 inference method (Huynh-Thu *et al.* 2010) was used together with GeneSNAKE here, without needing any prior integration into the package itself. For details, see the publication reproduction code for Fig. 3.

GeneSNAKE relies on a previously described ODE model (Marbach *et al.* 2010), while some other simulation tools have instead opted to rely partially or entirely on stochastic differential equations (SDEs) (Schaffter *et al.* 2011, Dibaeinia and Sinha 2020). We opted to only use ODEs as it gives a clear causality in the generated data before added noise, allowing for greater control of the noise level in the final data. This allows the user to fully customize the complexity of the problem by controlling the noise, as well as testing if a given model can, under no noise, solve the model perfectly. We note that for simulating single-cell data, SDEs may offer more biologically realistic models by better representing the heterogeneity between cells (Chen and Mar 2018). Despite this, we believe that for a benchmarking tool it is more important to be able to fully control the noise and complexity of the system, for which an ODE model is better suited.

We used GeneSNAKE to simulate data given a known GRN, and compared the time-dependent dynamics of the simulation with an experimental time series of gene expression. It was observed that GeneSNAKE data correlates well with experimental data, suggesting that the main trends of the experiment are accurately represented when generating data from known biological GRNs. This finding, alongside the flexible perturbation solution and noise models, highlights GeneSNAKE’s usefulness in not only generating data from simulated GRNs for benchmarking purposes but also to explore the behavior of experimental systems.

Depending on the type of data that is being modeled, GeneSNAKE implements different noise models. For instance, the log-normal noise model was previously shown to model microarray noise well, while the sequencing noise model is appropriate for scRNA-seq data. If the type of data is unknown, a general noise model based on multiplicative Gaussian noise is provided, and this is the default. Having multiple noise models in GeneSNAKE further adds value as it makes it possible to investigate how well an inference method performs for different types of noise.

Compared to other simulation packages such as GeneNetWeaver, an advantage of GeneSNAKE is that it gives much better control over GRN and data properties, and experimental design. Properties such as sparsity, stability, scale-freeness, condition number, variance, and noise level can have a different impact on different inference methods and therefore need to be varied in a controlled fashion. The GeneSPIDER package allows control of many of these properties but, like many other simulators and inference methods, simplifies the dynamics of gene regulation by only considering the steady-state, when the system no longer changes. Another issue with GeneNetWeaver is that it by default generates GRNs with link weights of 1, 0, or −1, which limits the quantitativeness of the system. With GeneSNAKE we combine the high controllability of GRN and data properties with time-dependent dynamical modeling as in GeneNetWeaver to obtain realistic yet flexible modeling. GeneSNAKE further has more advanced control of regulator collaboration, perturbation strength, and perturbation schemes.

Another important strength of the GeneSNAKE tool is the addition of FFL motifs in the network generation. These motifs have been shown to be enriched in biological GRNs (Milo *et al.* 2002, Lee *et al.* 2002, Burda *et al.* 2011) and have in previous benchmarks been shown to affect the performance of various models in different ways. For example, the large community-driven benchmark DREAM5 noted that even the best-performing regression models struggled to identify motif structures while information theory models could to a greater extent recover these motifs (Marbach *et al.* 2012). This hints at the importance of using realistic GRNs for simulation, as unrealistic GRN properties could lead to invalid performance comparisons of GRN inference methods, especially if they have different abilities to cope with motif biases.

While there are several advantages using realistic GRN structures and biochemical simulation models, several limitations arise from the chosen models. One of the key limitations that has already been touched upon is the assumption that FFL motifs play a key role in biological GRNs, and the increased difficulty in reconstructing FFL-enriched GRNs. While the enrichment is verified (Shen-Orr *et al.* 2002, Zhivkoplias *et al.* 2022), the complex dynamics of FFL motifs do make the GRN more complicated to reconstruct. This increased challenge in reconstruction risks reducing the usefulness of the simulated data in its role of benchmarking if all models perform equally poorly on the data, making it impossible to distinguish models. An example of this can be seen in the frequently cited DREAM5 (Marbach *et al.* 2012) challenge that compared GRNI methods on *in silico*, *Escherichia coli*, and *S. cerevisiae* data. They showed that while some methods performed well on *in silico* and *E. coli* data, performance on the more complex network derived from *S. cerevisiae* was so low that methods could not be separated.

Another key limitation of GeneSNAKE comes from the choice of using the established ODE model that makes up the backbone of GeneSNAKEs simulation model. While being a popular choice due to capturing biochemistry fairly well, it relies on simplifications of the true system. Chief among these simplifications is that it models a strict central dogma focused system, with all interactions going from DNA to protein via RNA and only proteins can have a regulatory function. Additional forms of regulation like chromatin state, post-transcriptional and translational regulation are not accounted for at all. Similarly all degradation of protein and RNA is assumed to be a linear function dependent on the λ parameter of a given gene or protein. A choice that for the model makes sense to minimize parameters but is likely not true in biological systems. In addition to this simplification the model comes with a large number of parameters that can be set. While most models opt to lock most of these we have here decided to leave them all open. This choice can make it intimidating to start experimenting with GeneSNAKE outside of the default parameters as the wrong choice can potentially cause the model to become unstable. Finally, due to the nature of ODE systems, simulations are computationally heavy making simulations of systems with thousands of genes highly time consuming.

We chose LSODA as the ODE solver even though it is known for being computationally heavy. The reason for this choice is that unlike most modern faster solvers, LSODA automatically handles the issue with stiff systems, i.e. systems that are difficult to solve because fast and slow effects happen simultaneously. This ability is advantageous when working with unknown GRN topologies as it prevents failures due to differences in the stiffness of the underlying GRN models. LSODA has been shown to be a good default choice for solving ODE systems with the main tradeoff coming from runtime (Städter *et al.* 2021).

## Supplementary Material

vbag039_Supplementary_Data

## Data Availability

The ENCODE data for HepG2 and K562 cell lines are publicly available via the ENCODE (ENCODE Project Consortium 2012) project website https://www.encodeproject.org/. See the publication reproduction code repository for a detailed description of its accession and processing. The data for the work by Hackett *et al.* 2020 is available at the IDEA project website https://idea.research.calicolabs.com/data. GeneSNAKE is publicly available as a Python package at: https://bitbucket.org/sonnhammergrni/genesnake/. Code for reproducing the figures and data processing in this publication is available at: https://bitbucket.org/sonnhammergrni/genesnake_publication_reproduction/.
