# Supplementary material for: GeneSNAKE: a Python package for simulation of gene regulatory networks and perturbation-induced expression data
Source: Bioinform Adv. 2026 Feb 6;6(1):vbag039. doi: 10.1093/bioadv/vbag039 (PMC13135626; doi:10.1093/bioadv/vbag039)
Supplement: vbag039_Supplementary_Data [file vbag039_supplementary_data.docx]

**Supplementary information**

GeneSNAKE: a Python package for benchmarking and simulation of gene regulatory networks and expression data

[**Supplementary text 2**](#_pi6oe9dskicl)

[1. Benchmarking 2](#_qghcuwscduk7)

[2. Sequencing noise model 2](#_mijxucoo7poq)

[3. GRN inference methods 3](#_s21q9poz3lgo)

[LASSO 3](#_1alezsv7lf2j)

[LSCO and LSCON 4](#_f46tvy69l7bt)

[Z-score 4](#_xp43ym2z4xtj)

[GENIE3 4](#_9q6imlin5exq)

[**Supplementary figures 6**](#_gv2i9wp7d9tt)

[**Supplementary references 9**](#_dcii0igyrfvp)

#

# **Supplementary text**

## **1. Benchmarking**

GeneSNAKE allows benchmarking of an inferred set of networks against a gold standard network. By comparing adjacency matrices it calculates the confusion matrix where true positives (TP), true negatives (TN), false positives (FP), and false negatives (FN) are summed up according to the presence of corresponding links. Afterward, the confusion matrix is used to estimate various performance measurements (Table S1) such as Area Under the Receiver Operating Characteristics (AUROC), Area Under the Precision-Recall (AUPR), sensitivity (TPR), specificity (TNR), precision (PPV), negative predictive value (NPV), miss rate (FNR), fall-out (FPR), false discovery rate (FDR), false omission rate (FOR), positive and negative likelihood ratio (LRP, LRN), prevalence threshold (PT), threat score (TS), F1-score, Matthew's correlation coefficient (MCC), Fowlkes-Mallows index (FM), informedness (BM), markedness (MK), and diagnostic odds ratio (DOR).

**Table S1.** Performance measurements implemented in GeneSNAKE for network benchmarking.

| measurement | | | | | | | | | |
| --- | --- | --- | --- | --- | --- | --- | --- | --- | --- |
| TPR | TNR | PPV | NPV | FNR | FPR | FDR | FOR | | LRP |
| $\frac{TP}{TP+FN}$ | $\frac{TN}{TN+FP}$ | $\frac{TP}{TP+FP}$ | $\frac{TN}{TN+FN}$ | $\frac{FN}{FN+TP}$ | $\frac{FP}{FP+TN}$ | $\frac{FP}{FP+TP}$ | $\frac{FN}{FN+TN}$ | | $\frac{TPR}{FPR}$ |
| measurement | | | | | | | | | |
| LRN | PT | TS | F1-score | MCC | FM | BM | MK | | DOR |
| $\frac{FNR}{TNR}$ | $\frac{\sqrt{FPR}}{\sqrt{TPR} +\sqrt{FPR}}$ | $\frac{TP}{TP+FN+FP}$ | $\frac{2*TP}{2*TP+FP+FN}$ | $\sqrt{PPV*TPR*TNR*NPV}$  -  $\sqrt{FDR*FNR*FPR*FOR}$ | $\sqrt{PPV*TPR}$ | $TPR+$  $TNR-1$ | $PPV+$  $NPV-1$ | | $\frac{LRP}{LRN}$ |

## **2. Sequencing noise model**

Inaccurately performed sequencing experiments may introduce a bias in gene expression measures [(Ross et al. 2013)](https://paperpile.com/c/wZyE83/KGxb). It may be caused by poorly designed experiments, GC-rich sequences, or various technical errors that lead to unmappable reads or over-mapping certain regions with reads. Furthermore, gene length is a factor that may affect the sequencing coverage. It has been concluded that short gene length bias was detected for scRNA-seq [(Phipson et al. 2017)](https://paperpile.com/c/wZyE83/zahr) similarly to bulk RNA-seq [(Oshlack and Wakefield 2009)](https://paperpile.com/c/wZyE83/HPRa).

To model synthetic data that follow the real data properties, we developed a coverage-based noise model. To achieve this, we imitate a sequencing process relying on the Lander-Waterman equation that allows us to estimate the sequencing coverage $C$ as follows: $C=\frac{L\times N}{G}$, where $L$ is the read length, $N$ is the total number of reads, and $G$ is the total length of a sequenced DNA. In this work, the values of $C$ and $L$ are assumed based on literature to set it according to a given sequencing technique, e.g. average coverage of 5 for RNA-seq studies [(McIntyre et al. 2011)](https://paperpile.com/c/wZyE83/WnTM). Moreover, sequencing error rates and gene lengths are also taken from literature or public databases. Some other parameters, such as the number of reads can be estimated based on the Lander-Waterman equation: $N=\frac{C\times G}{L}$.

To model the mapping procedure, we randomize the positions of aligned reads from a uniform distribution. Here, we take real gene start and end positions based on the human genome to establish their length. Afterward, we assume that the count value for a single gene in a given experiment is equal to the number of reads that cover the gene [(Pepke et al. 2009)](https://paperpile.com/c/wZyE83/nReU). This is referred to as simulated counts. Next, to simulate multiple experiments (or samples) this procedure is repeated a certain number of times. In addition, the entire process can be again repeated to simulate technical replicates. However, the described procedure simulates mainly subtractive noise and just a small fraction of additive noise from a GC-content-related step. Thus, the next stage of noise modeling involves the negative binomial (NB) distribution to simulate the distribution of counts as additive noise. Furthermore, zero inflation is introduced to data to simulate dropout rates. It is together known as a zero-inflated negative binomial (ZINB) distribution used in noise modeling for single-cell RNA-seq data [(Eraslan et al. 2019)](https://paperpile.com/c/wZyE83/zbQB). Finally, a matrix of error ratios is obtained by element-wise matrix operations as follows:

$E_{seq}={((counts}_{simulated} +E_{NB})⊘{counts}_{expected})\odot E_{ZI}$ (Eq. S1)

where ${counts}_{simulated}$ is a matrix of simulated counts, $E_{NB}$ is a matrix of erroneous counts drawn from the negative binomial distribution, ${counts}_{expected}$ is a matrix of theoretical counts estimated based on the Lander-Waterman equation for each gene, and $E_{ZI}$ is a binary matrix for zero inflation. To obtain a noise model with dropouts mimicking single-cell data, set the probability for $E_{ZI}$ greater than 0. Otherwise, set it to 0 to obtain dropout-free data mimicking bulk data. The error matrix is then used in a multiplicative way on noise-free data.

## **3. GRN inference methods**

#### LASSO

Least Absolute Shrinkage and Selection Operator (LASSO) is a widely used regularisation technique applying an L1 penalty to coefficient sizes in the loss function for regression model fitting, and the term was coined by Tibshirani in 1996 [(Tibshirani 1996)](https://paperpile.com/c/wZyE83/Zwv7).

van Someren et al. [(van Someren et al. 2002, 2006)](https://paperpile.com/c/wZyE83/Pi9S+A2Dd) and Inferelator [(Bonneau et al. 2006)](https://paperpile.com/c/wZyE83/5AFk) applied LASSO for GRN inference. van Someren claims in their 2006 paper [(van Someren et al. 2006)](https://paperpile.com/c/wZyE83/A2Dd) that their 2002 paper [(van Someren et al. 2002)](https://paperpile.com/c/wZyE83/Pi9S) was the first such application published. We made some attempts to verify this by looking for earlier occurrences, but the accessibility of the field literature starts declining around this time, due to incomplete literature digitalisation and reliance on conference papers.

Gardner et al. [(Gardner et al. 2003)](https://paperpile.com/c/wZyE83/P02M) and Julius et al. [(Julius et al. 2009)](https://paperpile.com/c/wZyE83/74UT) infers GRNs using a model structure with an explicit perturbation term.

Tjärnberg [(Tjärnberg et al. 2013)](https://paperpile.com/c/wZyE83/YaQS), combines the model structure of Gardner and Julius with LASSO for model fitting, and is the approach labeled LASSO in this publication.

The strength of the LASSO L1 parameter size penalty is configurable, and it was adjusted using Brent’s method to produce network sparsities of 50 links per gene on average.

#### LSCO and LSCON

The model used for Least Squares CutOff (LSCO) [(Tjärnberg et al. 2015)](https://paperpile.com/c/wZyE83/T1Ga) and LSCO with Normalisation (LSCON) [(Hillerton et al. 2022)](https://paperpile.com/c/wZyE83/bBbV) is

*Y = −A^−1^(P + F) + E*

*Y* is a log fold change steady state expression matrix after applying the perturbations *P*, and *A* is the interaction matrix or GRN. *E* is the expression matrix estimation noise, and *F* is the perturbation noise, the difference between intended and actual perturbation.

Both methods apply least squares regression using the Moore-Penrose generalised inverse, followed by a cutoff that eliminates weak (low absolute value) network links. LSCON additionally applies normalisation to avoid erroneous hyper connection of some genes, otherwise resulting from extreme values in the data.

#### Z-score

Z-score [(Prill et al. 2010)](https://paperpile.com/c/wZyE83/AFPF) estimates GRNs based on z-score normalisation of data. Regulatory links outgoing from some gene A are ranked based on the expression change of other genes when A is perturbed. This study includes two variants of Z-score. The original uses all observations to estimate gene expression mean and standard deviation. It is labeled Z-score. Z-score-P excludes perturbed observations of a gene from the estimation. This to avoid biasing the gene expression distribution with unrepresentative intentionally perturbed values.

#### GENIE3

GENIE3 [(Huynh-Thu et al. 2010)](https://paperpile.com/c/wZyE83/yYRa) constructs GRNs using random forest models. Each gene is predicted from the remaining, and the importances of genes for the prediction are used to construct a network. In contrast to the other methods in this study, GENIE3 does not explicitly utilise perturbation information, and instead works only with the expression data.

GENIE3 is not included in GeneSNAKE. The publication reproduction code therefore includes a separate setup of GENIE3.

The GRNs estimated by GENIE3 were transposed. Based on previously published results [(Seçilmiş et al. 2022)](https://paperpile.com/c/wZyE83/gQRd) and correspondence with the GENIE3 authors, when running GENIE3 on perturbation-based data, the results are substantially better when the predicted GRNs are transposed, i.e. the direction of regulator-target pairs are reversed.

# **Supplementary figures**

# **
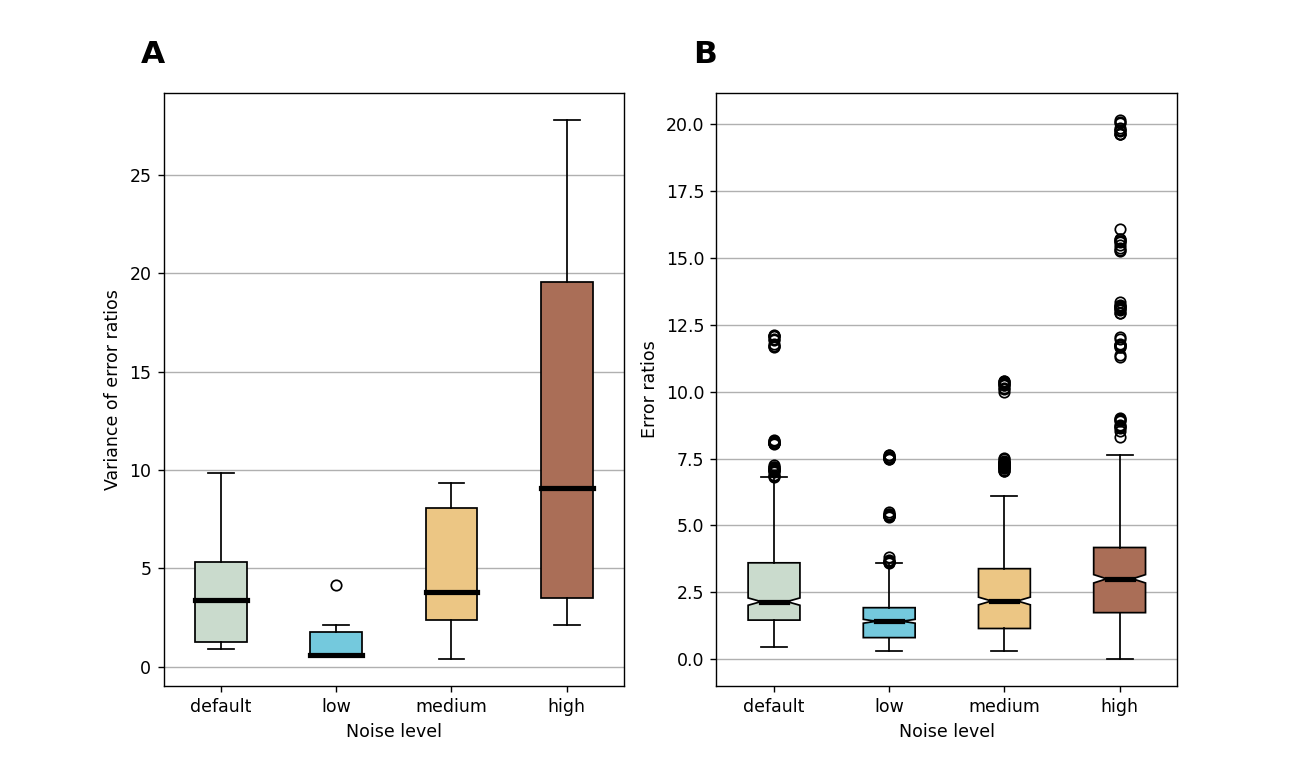
**

**Figure S1.** Performance of various noise options in sequencing-like noise by changing maximum alignment error (ermax), negative binomial probability (prob_nb), and variance scaling factor (sf). Some of the parameters were left default: minimum alignment error (ermin), technical replicates number (techrep), and probability of zero-inflation (prob_zi). The following settings were used: default (ermin=0.45, ermax=0.73, prob_zi=0, prob_nb=0.5, sf=0.75), low (ermax=0.6, prob_nb=0.6, sf=0.8), medium (ermax=0.7, prob_nb=0.5, sf=0.6) and high (ermax=0.8, prob_nb=0.4, sf=0.4) **A.** Variance of the error ratio matrix $E_{seq}$ and **B.** General statistics of the error ratio matrix $E_{seq}$. As ratios are randomly generated, each run was repeated six times. This analysis was performed for 10 randomly selected genes for a randomly generated count matrix of size 10x10.


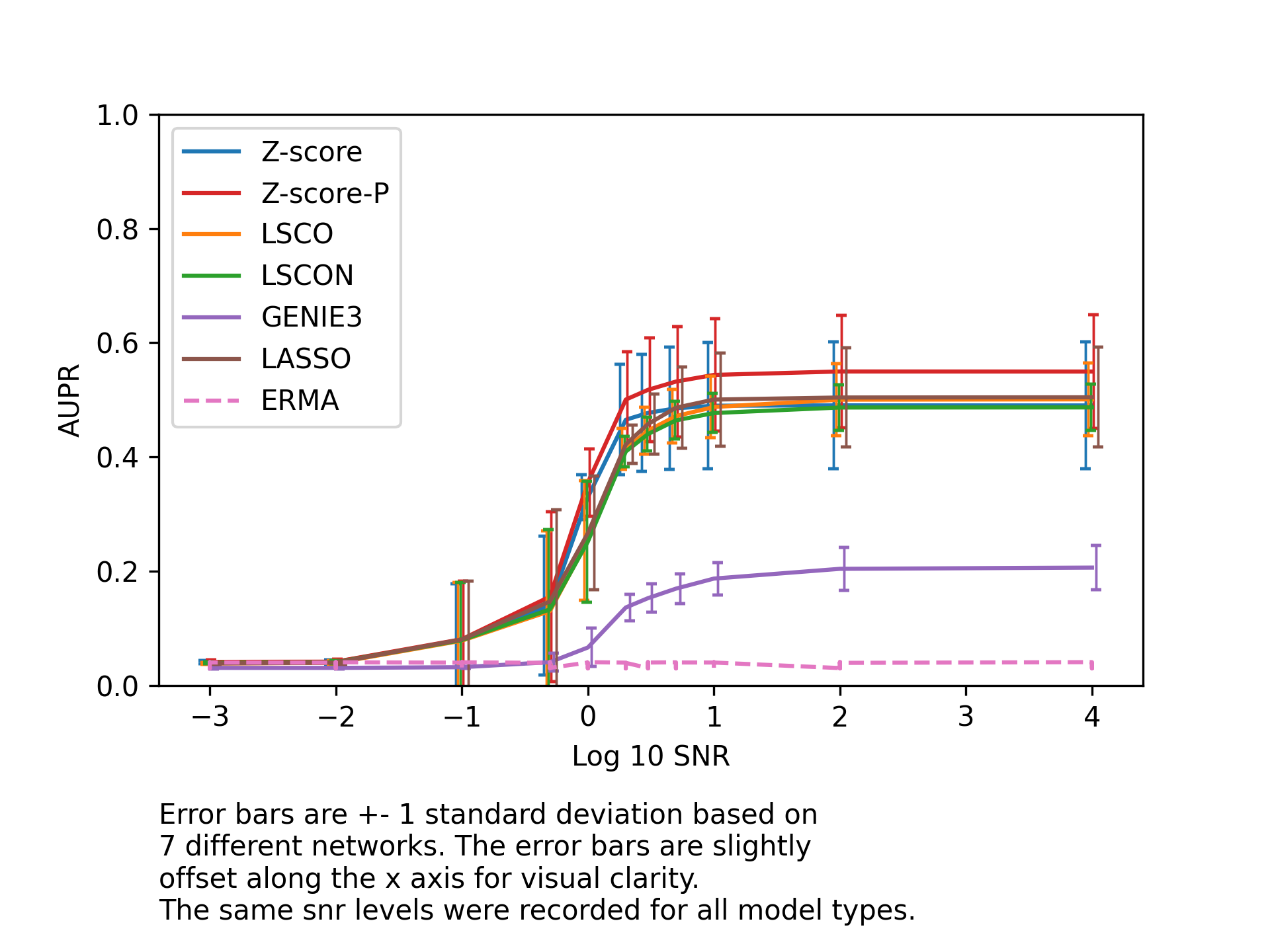

**Figure S2.** GRN inference accuracy against SNR level, using data produced by GeneSNAKE. Steady-state data with different Signal to Noise Ratio (SNR) levels was simulated from FFLatt GRNs containing 100 genes. Accuracy was measured in terms of Area Under Precision Recall (AUPR). Error bars are ± 1 standard deviation based on 7 different networks and data simulations. The error bars are slightly offset along the x-axis for visual clarity. The same SNR levels were recorded for all model types.


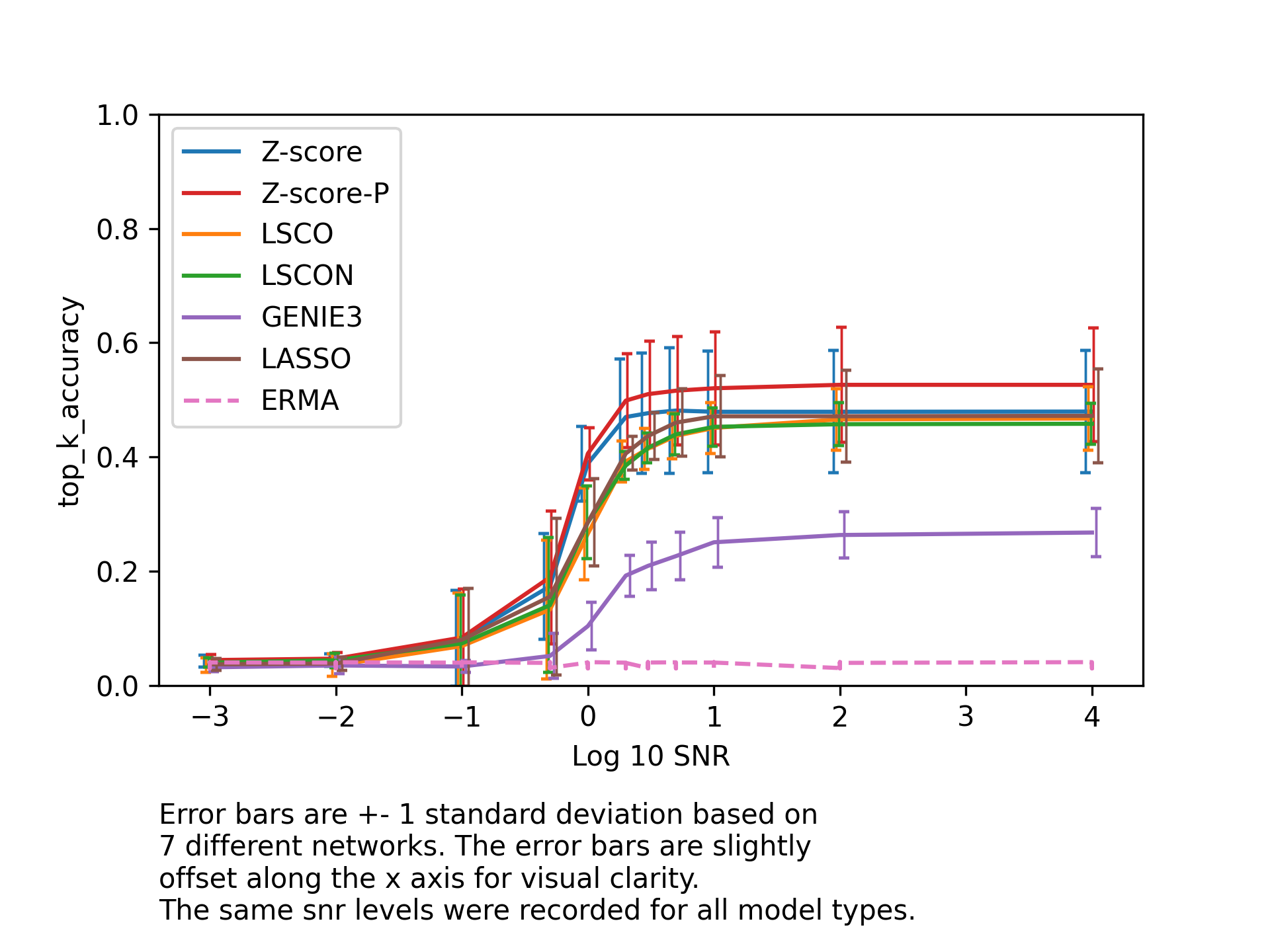


**Figure S3.** GRN inference accuracy against SNR level, using data produced by GeneSNAKE. Steady-state data with different Signal to Noise Ratio (SNR) levels was simulated from FFLatt GRNs containing 100 genes. Accuracy was measured in terms of top K accuracy, the accuracy of the top K most confidently predicted links, with K equalling the number of existing edges in the reference network. Error bars are ± 1 standard deviation based on 7 different networks and data simulations. The error bars are slightly offset along the x-axis for visual clarity. The same SNR levels were recorded for all model types.


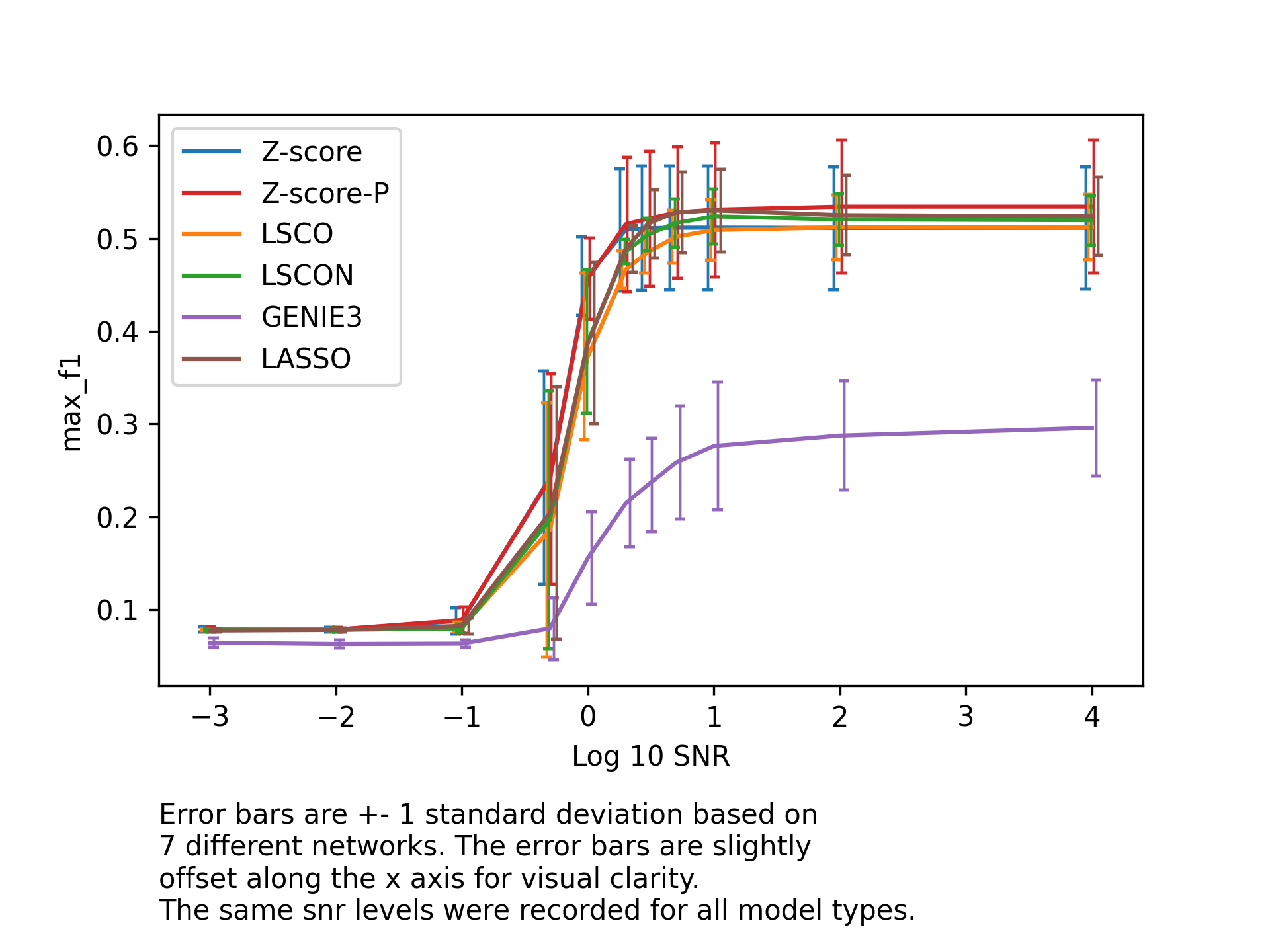


**Figure S4.** GRN inference accuracy against SNR level, using data produced by GeneSNAKE. Steady-state data with different Signal to Noise Ratio (SNR) levels was simulated from FFLatt GRNs containing 100 genes. Accuracy was measured in terms of maximum F1 score over thresholds for all values in the estimated networks. Error bars are ± 1 standard deviation based on 7 different networks and data simulations. The error bars are slightly offset along the x-axis for visual clarity. The same SNR levels were recorded for all model types.

# **Supplementary references**

[Bonneau, Richard, David J. Reiss, Paul Shannon, et al. 2006. “The Inferelator: An Algorithm for Learning Parsimonious Regulatory Networks from Systems-Biology Data Sets de Novo.” *Genome Biology* 7 (5): R36. https://doi.org/](http://paperpile.com/b/wZyE83/5AFk)[10.1186/gb-2006-7-5-r36](http://dx.doi.org/10.1186/gb-2006-7-5-r36)[.](http://paperpile.com/b/wZyE83/5AFk)

[Eraslan, Gökcen, Lukas M. Simon, Maria Mircea, Nikola S. Mueller, and Fabian J. Theis. 2019. “Single-Cell RNA-Seq Denoising Using a Deep Count Autoencoder.” *Nature Communications* 10 (1): 390. https://doi.org/](http://paperpile.com/b/wZyE83/zbQB)[10.1038/s41467-018-07931-2](http://dx.doi.org/10.1038/s41467-018-07931-2)[.](http://paperpile.com/b/wZyE83/zbQB)

[Gardner, Timothy S., Diego di Bernardo, David Lorenz, and James J. Collins. 2003. “Inferring Genetic Networks and Identifying Compound Mode of Action via Expression Profiling.” *Science (New York, N.Y.)* 301 (5629): 102–105. https://doi.org/](http://paperpile.com/b/wZyE83/P02M)[10.1126/science.1081900](http://dx.doi.org/10.1126/science.1081900)[.](http://paperpile.com/b/wZyE83/P02M)

[Hillerton, Thomas, Deniz Seçilmiş, Sven Nelander, and Erik L. L. Sonnhammer. 2022. “Fast and Accurate Gene Regulatory Network Inference by Normalized Least Squares Regression.” *Bioinformatics (Oxford, England)* 38 (8): 2263–2268. https://doi.org/](http://paperpile.com/b/wZyE83/bBbV)[10.1093/bioinformatics/btac103](http://dx.doi.org/10.1093/bioinformatics/btac103)[.](http://paperpile.com/b/wZyE83/bBbV)

[Huynh-Thu, Vân Anh, Alexandre Irrthum, Louis Wehenkel, and Pierre Geurts. 2010. “Inferring Regulatory Networks from Expression Data Using Tree-Based Methods.” *PloS One* 5 (9): e12776. https://doi.org/](http://paperpile.com/b/wZyE83/yYRa)[10.1371/journal.pone.0012776](http://dx.doi.org/10.1371/journal.pone.0012776)[.](http://paperpile.com/b/wZyE83/yYRa)

[Julius, A., M. Zavlanos, S. Boyd, and G. J. Pappas. 2009. “Genetic Network Identification Using Convex Programming.” *IET Systems Biology* 3 (3): 155–166. https://doi.org/](http://paperpile.com/b/wZyE83/74UT)[10.1049/iet-syb.2008.0130](http://dx.doi.org/10.1049/iet-syb.2008.0130)[.](http://paperpile.com/b/wZyE83/74UT)

[McIntyre, Lauren M., Kenneth K. Lopiano, Alison M. Morse, et al. 2011. “RNA-Seq: Technical Variability and Sampling.” *BMC Genomics* 12 (June): 293. https://doi.org/](http://paperpile.com/b/wZyE83/WnTM)[10.1186/1471-2164-12-293](http://dx.doi.org/10.1186/1471-2164-12-293)[.](http://paperpile.com/b/wZyE83/WnTM)

[Oshlack, Alicia, and Matthew J. Wakefield. 2009. “Transcript Length Bias in RNA-Seq Data Confounds Systems Biology.” *Biology Direct* 4 (April): 14. https://doi.org/](http://paperpile.com/b/wZyE83/HPRa)[10.1186/1745-6150-4-14](http://dx.doi.org/10.1186/1745-6150-4-14)[.](http://paperpile.com/b/wZyE83/HPRa)

[Pepke, Shirley, Barbara Wold, and Ali Mortazavi. 2009. “Computation for ChIP-Seq and RNA-Seq Studies.” *Nature Methods* 6 (11 Suppl): S22–32. https://doi.org/](http://paperpile.com/b/wZyE83/nReU)[10.1038/nmeth.1371](http://dx.doi.org/10.1038/nmeth.1371)[.](http://paperpile.com/b/wZyE83/nReU)

[Phipson, Belinda, Luke Zappia, and Alicia Oshlack. 2017. “Gene Length and Detection Bias in Single Cell RNA Sequencing Protocols.” *F1000Research* 6 (April): 595. https://doi.org/](http://paperpile.com/b/wZyE83/zahr)[10.12688/f1000research.11290.1](http://dx.doi.org/10.12688/f1000research.11290.1)[.](http://paperpile.com/b/wZyE83/zahr)

[Prill, Robert J., Daniel Marbach, Julio Saez-Rodriguez, et al. 2010. “Towards a Rigorous Assessment of Systems Biology Models: The DREAM3 Challenges.” *PloS One* 5 (2): e9202. https://doi.org/](http://paperpile.com/b/wZyE83/AFPF)[10.1371/journal.pone.0009202](http://dx.doi.org/10.1371/journal.pone.0009202)[.](http://paperpile.com/b/wZyE83/AFPF)

[Ross, Michael G., Carsten Russ, Maura Costello, et al. 2013. “Characterizing and Measuring Bias in Sequence Data.” *Genome Biology* 14 (5): R51. https://doi.org/](http://paperpile.com/b/wZyE83/KGxb)[10.1186/gb-2013-14-5-r51](http://dx.doi.org/10.1186/gb-2013-14-5-r51)[.](http://paperpile.com/b/wZyE83/KGxb)

[Seçilmiş, Deniz, Thomas Hillerton, Andreas Tjärnberg, Sven Nelander, Torbjörn E. M. Nordling, and Erik L. L. Sonnhammer. 2022. “Knowledge of the Perturbation Design Is Essential for Accurate Gene Regulatory Network Inference.” *Scientific Reports* 12 (1): 16531. https://doi.org/](http://paperpile.com/b/wZyE83/gQRd)[10.1038/s41598-022-19005-x](http://dx.doi.org/10.1038/s41598-022-19005-x)[.](http://paperpile.com/b/wZyE83/gQRd)

[Someren, E. P. van, B. L. T. Vaes, W. T. Steegenga, A. M. Sijbers, K. J. Dechering, and M. J. T. Reinders. 2006. “Least Absolute Regression Network Analysis of the Murine Osteoblast Differentiation Network.” *Bioinformatics (Oxford, England)* 22 (4): 477–484. https://doi.org/](http://paperpile.com/b/wZyE83/A2Dd)[10.1093/bioinformatics/bti816](http://dx.doi.org/10.1093/bioinformatics/bti816)[.](http://paperpile.com/b/wZyE83/A2Dd)

[Someren, E. P. van, L. F. A. Wessels, M. J. T. Reinders, and E. Backer. 2002. “Regularization and Noise Injection for Improving Genetic Networks Models.” In *Computational and Statistical Approaches to Genomics*. Kluwer.](http://paperpile.com/b/wZyE83/Pi9S) <https://research.tudelft.nl/en/publications/regularization-and-noise-injection-for-improving-genetic-networks/>[.](http://paperpile.com/b/wZyE83/Pi9S)

[Tibshirani, Robert. 1996. “Regression Shrinkage and Selection via the Lasso.” *Journal of the Royal Statistical Society. Series B, Statistical Methodology* 58 (1): 267–288. https://doi.org/](http://paperpile.com/b/wZyE83/Zwv7)[10.1111/j.2517-6161.1996.tb02080.x](http://dx.doi.org/10.1111/j.2517-6161.1996.tb02080.x)[.](http://paperpile.com/b/wZyE83/Zwv7)

[Tjärnberg, Andreas, Torbjörn E. M. Nordling, Matthew Studham, Sven Nelander, and Erik L. L. Sonnhammer. 2015. “Avoiding Pitfalls in L1-Regularised Inference of Gene Networks.” *Molecular bioSystems* 11 (1): 287–296. https://doi.org/](http://paperpile.com/b/wZyE83/T1Ga)[10.1039/c4mb00419a](http://dx.doi.org/10.1039/c4mb00419a)[.](http://paperpile.com/b/wZyE83/T1Ga)

[Tjärnberg, Andreas, Torbjörn E. M. Nordling, Matthew Studham, and Erik L. L. Sonnhammer. 2013. “Optimal Sparsity Criteria for Network Inference.” *Journal of Computational Biology: A Journal of Computational Molecular Cell Biology* 20 (5): 398–408. https://doi.org/](http://paperpile.com/b/wZyE83/YaQS)[10.1089/cmb.2012.0268](http://dx.doi.org/10.1089/cmb.2012.0268)[.](http://paperpile.com/b/wZyE83/YaQS)
